# PEDIATRIC LIVER TRANSPLANTATION WITH EX-SITU LIVER TRANSECTION AND THE APPLICATION OF THE HUMAN FIBRINOGEN AND THROMBIN SPONGE IN THE WOUND AREA

**DOI:** 10.1590/0102-6720201600040006

**Published:** 2016

**Authors:** Fernando Pompeu Piza VICENTINE, Adriano Miziara GONZALEZ, Ramiro Anthero de AZEVEDO, Barbara Burza BENINI, Marcelo Moura LINHARES, Gaspar de Jesus LOPES-FILHO, Jose Luiz MARTINS, Alcides Augusto SALZEDAS-NETTO

**Affiliations:** 1 Department of Surgical Gastroenterology and Liver Transplantation; 2 Postgraduation in Interdisciplinary Surgical Science, Federal University of São Paulo - UNIFESP, São Paulo, SP, Brazil

**Keywords:** Liver transplantation. Hemostasis. Hepatectomy, Techniques

## Abstract

**Background:**

Surgical strategy to increase the number of liver transplants in the pediatric population is the ex-situ liver transection (reduction or split). However, it is associated with complications such as hemorrhage and leaks. The human fibrinogen and thrombin sponge is useful for improving hemostasis in liver surgery.

**Aim:**

Compare pediatric liver transplants with ex-situ liver transection (reduction or split) with or without the human fibrinogen and thrombin sponge.

**Methods:**

Was performed a prospective analysis of 21 patients submitted to liver transplantation with ex-situ liver transection with the application of the human fibrinogen and thrombin sponge in the wound area (group A) and retrospective analysis of 59 patients without the sponge (group B).

**Results:**

The characteristics of recipients and donors were similar. There were fewer reoperations due to bleeding in the wound area in group A (14.2%) compared to group B (41.7%, p=0.029). There was no difference in relation to the biliary leak (group A: 17.6%, group B: 5.1%, p=0.14).

**Conclusion:**

There was a lower number of reoperations due to bleeding of the wound area of ​​the hepatic graft when the human fibrinogen and thrombin sponge were used.

## RESUMO

**Racional:**

Estratégia cirúrgica para aumentar o número de transplantes hepáticos na população pediátrica é a transecção hepática ex-situ (redução ou split). No entanto, ela está associada com complicações, tais como hemorragia e fístulas. A esponja de fibrinogênio e trombina humana é útil para melhorar a hemostasia nas operações hepáticas.

**Objetivo:**

Comparar transplantes hepáticos pediátricos com transecção hepática ex-situ (redução ou split) com ou sem a esponja de fibrinogênio e trombina humana.

**Métodos:**

Foi realizada análise prospectiva de 21 pacientes submetidos ao transplante de fígado com transecção hepática ex-situ com a aplicação da esponja de fibrinogênio e trombina humana na área cruenta (grupo A) e análise retrospectiva de 59 pacientes sem a esponja (grupo B).

**Resultados:**

As características dos receptores e doadores eram semelhantes. Observou-se menor número de reoperações devido à hemorragia na área da cruenta no grupo A (14,2%) em comparação com o grupo B (41,7%, p=0,029). Não houve diferença em relação à fístula biliar (grupo A: 17,6%, grupo B: 5,1%, p=0,14).

**Conclusão:**

Houve menor número de reoperações por sangramento da área cruenta do enxerto hepático quando a esponja de fibrinogênio e trombina humana foi utilizada.

## INTRODUCTION

Pediatric liver transplantation needs to deal with the difficulty of finding small deceased donors in whom the hepatic graft fits the size of the recipient^7^ ^,^ ^20^ ^,^ ^24^ and organ shortage is a problem described by liver transplant teams, making the mortality in the awaiting list a reality.

As alternatives to small recipient cases - in an attempt to increase the supply of liver grafts - techniques were developed, such as liver transplantation with ex-situ liver transection of the graft from the deceased donor. When talking about liver transplantation with ex-situ liver transection, these are two possible procedures: hepatic reduction or split^17^ ^,^ ^26^ ^,^ ^30^.

Hepatic reduction, described by Bismuth et al. in 1984^4^, a non ruler hepatectomy is performed in the hepatic graft (where the hepatic resection line does not preserve the hepatic anatomical segments^33^) during the preparation of the organ at the auxiliary table, in order to reduce the size of the graft to fit the size of the recipient.

**FIGURE 1:**
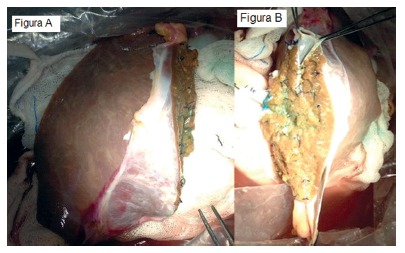
Hepatic graft submitted to split with formation of two functional liver grafts: A) graft for the adult; B) graft for the child

The split technique was described by Pichlmayr in 1988^27^, in which, from a cadaveric donor the liver graft is divided, into an auxiliary table, respecting the anatomic liver segments^33^ and preserving the vascular structures, obtaining two partial grafts able to transplant for two different recipients (Figure 1). A graft composed of segments II and III (used in a pediatric receptor) and another by segments I, IV-VIII (used in an adult recipient).

Currently, the ex-situ liver transection technique achieves good results in the literature, with survival rates similar to the results of transplants with whole graft^13^ ^,^ ^20^ ^,^ ^23^ ^,^ ^24^ ^,^ ^30^, but its use is not free of complications such as increased bleeding of the wound area of the graft, with greater use of blood products and biliary leak^14^ ^,^ ^23^ ^,^ ^30^ ^,^ ^36^.

The human fibrinogen and thrombin sponge (Tachosil(r))^16^ is a material to aid in surgical hemostasis, consisting of a matrix of collagen associated with a layer of plasma components such as coagulation factors, fibrinogen and fibrin. Its use in liver surgery was described in studies, showing that after hepatectomy its use obtained fast and effective hemostasis in the wound area when compared to other methods^5^ ^,^ ^12^ ^,^ ^14^ ^,^ ^29^. New articles describe the use of sponge in the field of liver transplantation with good results in relation to hemostasis and biliary leak^21^ ^,^ ^36^.

The objective of this study was to compare pediatric liver transplantation with the ex-situ liver transection with or without the use of the human fibrinogen and thrombin sponge.

## METHODS

The project was approved by the Research Ethics Committee of the Federal University of São Paulo/Hospital São Paulo, with number 38311/2012.

A prospective study of pediatric patients submitted to liver transplantation with the ex-situ liver transection technique and with the use of the human fibrinogen and thrombin sponge was carried out at Hospital São Paulo, Escola Paulista de Medicina - UNIFESP, São Paulo, SP, Brazil, from January 2012 to May 2016. The control group was formed by a historical cohort, with prospective data collection, starting in January 2004, of all patients submitted to liver transplantation with ex-situ liver transection technique without the use of the human fibrinogen and thrombin sponge in this same service.

The study included 80 patients and divided into two groups: the first, classified as group A, was composed of 21 patients submitted to the transplantation with the use of the human fibrinogen and thrombin sponge; and in the control group (group B) were included 59 patients submitted to the transplantation without the use of fibrinogen sponge and human thrombin.

Inclusion criteria were children of up to 18 years of age, of both genders, submitted to liver transplantation with ex-situ liver transection technique independent of indications of liver transplantation. Patients older than 18 years and cases in which the parents or guardians refused to sign the free and informed consent form were excluded.

Potential donors were selected for hepatic transplantation with the ex-situ liver transection technique when, if possible, they fit within the criteria of this institution: under 50 years of age, hemodynamic stability, less than four days in the intensive care, ALAT (U/l) and ASAT (U/l) levels less than twice normal, GGT below 50 IU/l, sodium levels below 160 mmol/l and less than 30% fat infiltration In the macroscopic analysis. Due to the severity of the recipients and the urgency of a liver graft for transplantation, not all donors met these criteria.

Aiming for the most appropriate matching between donor and recipient, the size, weight and BMI data of both were taken into account in the decision to perform the liver transection. The logistics between liver graft extraction, liver transection (reduction or split) on an auxiliary table, and graft implantation were designed to achieve maximum cold ischemia time of 12 h.

After liver graft revascularization, primary hemostasis and raffia of the bile ducts of the wound area were performed. According to the manufacturer's instructions, the previously moistened sponge and was applied the yellow side, on which the active principles of the product were found, to the wound surface of the liver graft. Sponge compression was carried out with the aid of a wet compress for about 2 min in order to obtain a correct sponge fixation and local hemostasis.

**FIGURE 2:**
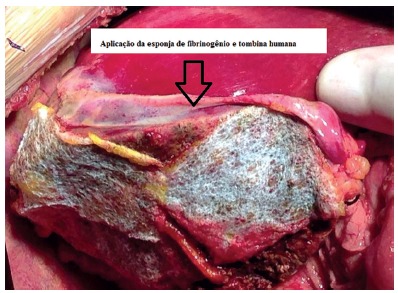
Liver graft submitted to split using human fibrinogen and thrombin sponge in wound area

When necessary, because of the extent of the wound area being larger than the surface of the sponge, more than one was used and the application process was repeated (Figure 2).

The outcomes evaluated in this study were: use of blood products, bleeding of the wound area, biliary leak and collection of the wound area, and survival of the patient and liver graft in 30 days.

### Statistical analysis

The analyzed data are represented in mean, median, standard deviation and percentage. Student's t-test, chi-square, Fisher exact, Mann-Whitney and Kaplan Meier survival curve were applied when indicated. The statistical analysis program SPSS version 2.0 was used, with p <0.05 being considered significant.

## RESULTS

The two groups were similar in terms of demographic characteristics, including age, gender, diagnosis of liver disease, weight, height, blood typing and PELD range.

In relation to the donors, except for the level of bilirubin (mg/dl), which was higher in group A (0.71±0.55) than in group B (0.47±0.37), but both above the normality, there was no difference in the other characteristics and both group were similar to age (years), gender, body mass index (BMI), use of inhaled drugs, alcohol consumption (mg/dl), creatinine (mg/dl), urea (mg/dl), ALAT (U/l), ASAT (U/l), bilirubin (mg/dl), alkaline phosphatase (U/l) and GGT (U/ l).

Group A consisted of 18 (85.7%) patients submitted to the split technique and three (14.3%) to the reduction technique; in group B, 36 (61%) patients were submitted to the split technique and 23 (39%) to the reduction technique (p=0.056).

The parameters analyzed in the surgical procedure (Table 1) showed that both groups received red blood cell transfusion, and the median use of blood products (ml/kg) was 35.1 in group A and 36.5 in group B, without statistically significant difference (p=0.83).

**TABLE 1:** Liver transplantation data

	Group A (n = 21) With sponge	Group B (n = 59 Without sponge	p
Use of red blood cells	100 %	100 %	1
Hemoderivative (ml/kg)	35,1	36,5	0,83
Reoperation due to bleeding of the wound area	14,2%	41,7%	0,029
Collections	14,28%	9,75%	0,68
Bile leak of the wound area	5,88%	5,12%	0,87

When the reoperations by bleeding of the wound area were analyzed, there was a lower frequency in group A (14.2%) than in B (41.7%), with a statistically significant difference (p=0.029).

Regarding the biliary leak and collection of the wound area, as well as the type of treatment indicated for the collection, such as reoperation, puncture and drainage of the collections, there was no statistical difference when comparing the two groups.

Regarding the early survival in 30 days, comparing the two groups, group A presented survival of 80.9% in 30 days and the B 87.7%, with no statistically significant difference (p=0.328). Early 30-day survival of liver grafts was also similar, comparing the two groups (group A: 76.1% and group B: 78.4%, p=0.668).

## DISCUSSION

The two groups studied did not present statistical differences regarding the demographic characteristics of the donors and recipients, forming two groups similar and comparable for possible outcomes. The techniques of ex-situ liver transection were similar in reduction and split.

An alternative to the ex-situ split technique would be the use of split in-situ, in which the liver graft is divided with the donor still the heart beating. This technique has already been described in the literature^1^ ^,^ ^6^ ^,^ ^11^ ^,^ ^18^ ^,^ ^28^ ^,^ ^35^, and it is known that there is an increase of about 2 h during the harvesting operation when employed^10^ ^,^ ^28^, in addition to the need for adequate equipment to perform this procedure in the hospital where the capture is performed.

These conditions are difficult to find in Brazil due to their extension and non-standardization of health services, so this technique has been little used in the country. The Pittisburgh transplant group published a comparison between in-situ and ex-situ split techniques in 2000, showing no difference in patient survival and hepatic graft survival^28^.

Hemotransfusion frequency was observed in 100% of patients in both groups during liver transplantation, red blood cell transfusion volume was statistically similar, and the median use of blood products (ml/kg) was 35.1 in group A and 36.5 in group B, with no statistically significant difference (p=0.83).

In a study published in 2010, Toti el al. compared the use of human fibrinogen and thrombin sponge with fibrin glue in the wound area of ​​patients submitted to liver transplantation with the ex-situ split technique. The authors described no difference in the need for the use of blood transfusion between the two groups, but did not demonstrate data of transfusion at work and only cited this information during the text^36^.

When the reoperations by bleeding of wound area were analyzed we observed that in group A - where the human fibrinogen and thrombin sponge were applied - the reoperation rate was lower (14.2%) than in the Group B (41.7%, p=0.029), demonstrating that this instrument may help in the hemostasis of the wound area of patients submitted to pediatric liver transplantation with the ex-situ liver transection technique (reduction or split).

In the literature, articles involving the use of this sponge for hemostasis of the wound area in hepatectomy and in liver transplants with the ex-situ liver transection technique and/or living donor, corroborate this finding^5^ ^,^ ^12^ ^,^ ^14^ ^,^ ^21^ ^,^ ^25^ ^,^ ^29^ ^,^ ^36^.

In 2008 a traditional pediatric transplant group in Belgium published a report of the use of human fibrinogen and thrombin sponge in four children undergoing liver transplantation using the liver transection technique (two received the left segment of alive donor and two a reduced hepatic graft). However, it was a series of cases without a control group and the author's opinion is that the human fibrinogen and thrombin sponge used on the wound area of the graft leads to a good and effective hemostasis, which occurs by describing the use of only one concentrate of red blood cells per patient during the transplantation, as no patient presented bleeding in the postoperative period^21^.

In 2011 Mirza et al. published a prospective and multicenter study analyzing the use of the human fibrinogen and thrombin sponge in a population composed of individuals submitted to liver transplantation with the ex-situ liver transection technique and others to hepatectomy. Sixteen patients were included, 13 of whom underwent liver transplantation. The authors demonstrated good hemostasis of the wound area with the use of the human fibrinogen and thrombin sponge within 3 min in 81% of the patients studied^25^. An interesting point was the way of conducting the study, since the population planned for the analysis was 40 patients. However, during the study, it was interrupted due to the fact that, as the authors wrote, it was evident that the data collected were related to the underlying liver disease or to the surgical procedure, and not to the use of the human fibrinogen and thrombin sponge^25^.

In this study, there was no statistically significant difference in the biliary leak rate of the wound area, as well as in the collection area near the wound area between the group that used the sponge (5.88% and 14.28%, respectively), and the group that did not used the sponge (5.12% and 9.75%, respectively, p=0.87 and p=0.68, respectively).

Biliary leak in hepatic transplantation is an important cause of morbidity. Literature rates vary from 5-10% of liver transplants with whole liver and are directly related to complications of the hepatic artery^9^ ^,^ ^16^ ^,^ ^22^ ^,^ ^31^. In cases of liver transplantation with the ex-situ liver transection technique (reduction or split), these rates may increase^9^ ^,^ ^10^ to 18.8%, as described by Diamond et al. in the year 2007^8^.

In the literature, the use of human fibrinogen and thrombin sponge and its relation with biliary leak and collection of the wound area is still controversial.

Toti et al., in a retrospective study in 2010 demonstrated a reduction in the biliary leak rate in adult patients submitted to liver transplantation with split and sponge use in the wound area compared to fibrin glue use in the wound area, reducing the rate of leak of 43.75% without the use of the sponge, to 6.25% with its use^36^.

On the other hand, Mirza et al. demonstrate a rate of 12.5% of reoperation due to bile leak of the wound area in pediatric patients submitted to liver transplantation even with the use of the human fibrinogen and thrombin sponge in the wound area^25^.

It was studied the early survival in 30 days, because after this period the human fibrinogen and thrombin sponge has been absorbed and is no longer present in the patients' bodies. Both groups presented 30-day mortality and graft loss in 30 days similar, with no statistically significant difference. Patient survival at 30 days in group A was 80.9% and in B, 87.7% (p=0.32). The graft survival in group A was 76.1% and in B, 78.4% (p=0.668). Regarding patient and graft survival in 30-day period, the values are similar to those presented by the other authors in the literature. The one-year survival of pediatric patients undergoing liver transplantation with the ex-situ liver and graft transection technique is described in the literature in about 73% and 63%^26^, and may reach 91% and 90% in a highly specialized center. A review article on liver transplantation with the split technique, published in 2003, presented data from authors with rates referring to one-year survival of patients and grafts ranging from 76% to 100% and 66% to 100%^20^.

## CONCLUSION

Comparing pediatric liver transplantation with the ex-situ liver transection technique (reduction or split) with or without the use of the human fibrinogen and thrombin sponge, was observed a smaller number of reoperations due to bleeding of wound area of the liver graft when the human fibrinogen and thrombin sponge were used.
